# A population-based study of health-promoting behaviors and their predictors in Iranian males, 2019

**DOI:** 10.1186/s13690-021-00543-1

**Published:** 2021-02-25

**Authors:** Fovziye Sanaati, Mehrnaz Geranmayeh, Zahra Behboodi Moghadam, Armin Zareiyan, Keshvar Samadaee Gelehkolaee, Mojgan Mirghafourvand

**Affiliations:** 1 Department of Midwifery, Faculty of Nursing and Midwifery, Tehran University of Medical Sciences, Tehran, Iran; 2 School of Nursing & Midwifery Tehran University of Medical Sciences, Tehran, Iran; 3 Public Health Department, Nursing Faculty, AjA University of Medical Sciences, Tehran, Iran; 4 Midwifery Department, Social determinants of Health Research Center, Tabriz University of Medical Sciences, P.O. Box: 51745-347, Shariati Street, Tabriz, 513897977 Iran

**Keywords:** Health-promoting behaviors, Social support, Sociological features, Men

## Abstract

**Background:**

Health-promoting lifestyle (HPL) is any measure taken to maintain a person’s health. The most important and influential factor in maintaining and enhancing health are health-promoting behaviors (HPB). This study aimed to determine HPB and their predictors among Iranian men.

**Methods:**

In this cross-sectional study, 783 Iranian men, living in Tehran, were selected, using multistage cluster sampling. The employed questionnaires, namely the sociodemographic questionnaire, Health-Promoting Lifestyle Profile II (HPLP-II), and the second part of the Personal Resource Questionnaire (PRQ 85-Part 2), were completed through interviews. The relationship between the dependent variables (HPLP-II and its subdomains) and independent variables (sociodemographic characteristics and social support) was investigated using the adjusted General Linear Model (GLM).

**Results:**

The mean ± standard deviation of the total HPLP-II score was 2.72 ± 0.44 in the range of 1–4. Among the six dimensions of the HPB, the participants achieved the highest score (3.00 ± 0.52) and lowest score (1.96 ± 0.56) in spiritual growth and physical activity, respectively. The Pearson test showed that the perceived social support was significantly correlated with HPLP-II (r = 0.23; *p* < 0.001) and all of its subdomains (*r* = 0.09–0.24; *p* < 0.001). Based on the adjusted general linear model, social support, age, job, and income adequacy were the predictors of HPL in men and could explain 30.9% of the variance of the HPL score.

**Conclusions:**

The research findings confirmed the importance of social support and modifying variables (social and personal) in the incidence of HPB in men.

## Background

Health promotion is a process that empowers people to increase control over and to improve their health [1]. The major focus of health promotion is on disease prevention and self-care ability [2]. The health-promoting behaviors (HPB(are among the most determinant health factors and plays a major role in disease prevention [3–6]. These behaviors not only prevent diseases but also improve the patients’ quality of life and life expectancy [7–10]. On the other hand, failure to adopt these behaviors has a direct correlation with the development of diseases [11] and mortality [8].

Pender (1996) divided the HPB into six dimensions of nutrition, physical activity, stress management, health responsibility, interpersonal relationship, and spiritual growth [12]. In other words, the health promotion is comprised of certain behaviors by which a person engages with proper nutrition, regular exercise, avoidance of destructive behaviors, protection against accidents, early diagnosis of disease symptoms, control of emotions and feelings, adaptation with stress and problems, and improvement of interpersonal relationships [13]. Studies have shown that inappropriate health behaviors, lack of health-seeking behaviors, and self-neglect are more common among men than women [14, 15]. Unhealthy behaviors among men, such as alcohol consumption, tobacco smoking, and poor nutrition and its subsequent overweight/obesity, are directly correlated with such diseases as high blood pressure in the future [16]. On the other hand, these three risk factors pose the men’s disability-adjusted life year (DALYs) to risks and increase the incidence of mortality and morbidity among them [17].

Studies have been conducted to investigate HPB in different countries and among different groups, including adolescents [13, 18], women of reproductive age [19], men [20], students [21], medical staff [22], and migrant workers [23]. The observed differences in HPB can be due to determinant health factors, such as individual, social, and economic factors, national health policies, mass media, advertisement methods, and living environment [20]. Another major health factor is social support, which has a significant direct relationship with health promotion and its associated behaviors [24–26]. Social support is defined as a subjective feeling of attachment, acceptance, being loved, and receiving help when needed [27].

The HPB and their relationship with perceived social support were investigated among Iranian women at reproductive age [19]. Despite the men’s lifestyle issues, this subject has not been studied in Iran. Given many challenges concerning male health, the difference between male’s lifestyle and female’s lifestyle, and cultural, social, economic, and environmental differences in Iran, it is necessary to address this subject. The results may be useful in making intervention plans for health promotion in this important population group, resulting in their lifestyle quality enhancement. Therefore, this study aimed to find out HPB among men and investigate the relationship of perceived social support and socioeconomic variables with these behaviors among men.

## Methods

### Study design and participants

This cross-sectional population-based survey with a sample size of 783 men was conducted in Tehran in 2019.

The inclusion criteria were Iranian nationality, living in Tehran, and age group of 15–65 years. The exclusion criteria were having severe mental disorder and lack of speech ability which inhibited responding to the questions.

### Sampling

The participants were selected using multistage cluster sampling method. Tehran (capital of Iran) has 22 municipality districts. Each municipality district of Tehran includes a number of regions (generally 300 regions(, and each region includes a number of blocks. First, 50 regions were selected from all 22 districts of Tehran, using the probability sampling method (proportional to the number of households in each district) and then one block was selected randomly from each region. Next, 15 households were selected from each block using systematic sampling. Data collection was done by four trained interviewers through in-person interviews in the evening when the participants were more likely to be at home. Before recruitment to the study, all participants were provided with information about the nature and aim of the study. Moreover, the informed consent of all participants was obtained. In this study, there were not any dropped-out and all of 783 participants finalised the interview.

### Questionnaires

Quantitative technique was applied for data collection. Data collection instruments included sociodemographic questionnaire, Health-Promoting Lifestyle Profile II (HPLP-II), and the second part of the Personal Resource Questionnaire (PRQ 85-Part 2).

The sociodemographic information included age, marital status, educational attainment, job, income adequacy, and homeownership status.

The HPLP-II included six categories, namely health responsibility, physical activity, nutrition, spiritual growth, stress management, and interpersonal relationships. It has 52 four-choice items scored by 1 “never,” 2 “sometimes,” 3 “often,” and 4 “always.” The total HPL score was between 52 and 208. The HPLP-II was validated in Iran with an estimated total Cronbach’s alpha of 0.82. The Cronbach’s alpha for its six dimensions ranged between 0.64–0.91 [28]. In this study, the Cronbach’s alpha and ICC of the HPLP-II were 0.89 and 0.92, respectively. The Cronbach’s alpha and ICC of its subdomains varied in the range of 0.67–0.78 and 0.78–0.89, respectively.

The PRQ 85-Part 2 questionnaire included 25 7-point Likert scale items, anchored by “completely agree” to “completely disagree” scored in the range of 25–175 [29]. In this study, the Cronbach’s alpha and ICC of this instrument were 0.91 and 0.88, respectively.

The HPLP-II and PRQ 85-Part 2 were translated into Farsi in Iran and their validity and reliability were assessed in pilot studies [28, 30].

### Sample size and data analysis

The estimated sample size was 494 using G-power, based on the results from Mirghafourvand et al.’s study (19), and the correlation coefficient of 0.12 between social support and physical activity of HPB, α = 0.05, power = 85%. The final sample size was 749 because of using cluster sampling and a design effect of 1.5. However, the sample size was ultimately increased to 783 by considering a 5% sample loss.

Data were analyzed with SPSS26. The sociodemographic characteristics, HPB, and social support were described using descriptive statistics including frequency, percent, mean, and standard deviation. The Pearson correlation was used to investigate the relationship between HPB and perceived social support. The one-way ANOVA, Pearson correlation, and independent t-test were used to determine the relationship between sociodemographic characteristics and HPB. In the end, the adjusted general linear model was used to predict the effect of each independent variable (perceived social support and demographic characteristics) on the dependent variable (HPLP-II and its subdomains) and also to determine the variance.

## Results

### Sociodemographic properties

Approximately half the participants (48.02%) were in the range of 25–34 years with the mean age of 38.1 years. The majority of the participants were married (69.85%). About two-thirds of the participants (64.1%) were freelancers. In addition, nearly two-thirds of the participants (60.40%) had a high-school diploma or an academic degree. Approximately, 45% of the participants reported that their monthly income was somewhat sufficient (Table 1).

**Table 1:** Sociodemographic characteristics of the participants (Iranian men), 2019 (*n* = 783)

Characteristics	n (%)
**Age** (years)	
15–24	103 (13.15)
25–34	376 (48.02)
35–65	304 (38.82)
**Marital status**	
Single	134 (17.11)
Married	547 (69.85)
Divorced	90 (11.49)
Widowed	12 (1.53)
**Occupation**	
Worker	37 (4.72)
Experts/managers	83 (10.60)
Shopkeeper	185 (23.62)
Private sector	478 (61.04)
**Sufficiency of income for expenses**	
Completely	369 (47.12)
To some extent	349 (44.57)
Absolutely not	65 (8.30)
**Home**	
Personal	238 (30.39)
Rent	545 (69.60)
**Education**	
Illiterate	13 (1.66)
Elementary school	28 (3.57)
Secondary school	125 (15.94)
Some high school	144 (18.39)
High-school diploma	249 (31.80)
University	224 (28.60)

### Health-promoting behaviors and perceived social support

The mean ± standard deviation of the total HPLP-II score was 2.72 ± 0.44 within the score range of 1–4 (score 1 is the worst and score 4 is the best condition in terms of HPB). This finding showed that Iranian men moderately adopt HPB. The participants obtained the highest scores in spiritual growth (3.01 ± 0.52), interpersonal relationships (2.88 ± 0.55), nutrition (2.88 ± 0.47), and health responsibility (2.87 ± 0.69). The lowest score was obtained in physical activity (1.96 ± 0.55) and the moderate score in stress management (2.58 ± 0.56) (Table 2). The mean ± standard deviation score of the perceived social support was 126.30 ± 40.26 within the range of 25–175.

**Table 2:** Mean and standard deviation for perceived social support, health-promoting lifestyle profile II (HPLP-II) and subscales and their association with perceived social support in Iranian men, 2019

Variable	Mean (SD)	Obtained score range	Obtainable score range	Relationship with social support r (*P*) *
HPLP-II	2.72 (0.44)	1.44–3.79	1–4	0.23 (< 0.001)
Nutrition	2.88 (0.47)	1.67–4.00	1–4	0.24 (< 0.001)
Physical activity	1.96 (0.56)	1.00–4.00	1–4	0.09 (0.011)
Spiritual growth	3.01 (0.52)	1.50–4.00	1–4	0.24 (< 0.001)
Health responsibility	2.87 (0.69)	1.44–4.00	1–4	0.18 (< 0.001)
Interpersonal relations	2.88 (0.55)	1.56–4.00	1–4	0.21 (< 0.001)
Stress management	2.58 (0.56)	1.29–4.00	1–4	0.12 (< 0.001)
Social support	1.26.30 (40.26)	25.00–175.00	25–175	

### Relationship of health-promoting behaviors and perceived social support

Based on the Pearson correlation test, the total HPLP-II score (correlation coefficient (r) = 0.23; *p* < 0.001) and its subdomains, namely nutrition (*r* = 0.24; *p* < 0.001), physical activity (*r* = 0.24; *p* < 0.001), health responsibility (*r* = 0.18; *p* < 0.001), interpersonal relationships (*r* = 0.21; *p* < 0.001), and stress management (*r* = 0.12; *p* < 0.001) were significantly correlated with the social support (Table 2). The results of bivariate tests (one-way ANOVA, Pearson correlation, and independent t-test) showed that there was a significant relationship between age, homeownership status, income sufficiency, and job with HPB. These variables, along with social support were inputted into the general linear model. Social support, age, job and income sufficiency were the predictors of HPB in men and could explain 30.9% of the variance in the HPB score (Table 3).

**Table 3:** The general linear model for factors related to the overall score of health-promoting lifestyle profile II (HPLP-II) in Iranian men, 2019

Variable	B (95% Confidence Interval)	P*
**Social support**	−0.001 (− 0.002 to 0.000)	0.025
**Age**	0.008 (0.005 to 0.012)	< 0.001
**Job**		
Worker	−0.07 (− 0.201 to − 0.050)	0.241
Experts/managers	0.114 (0.026 to 0.202)	0.011
Shopkeeper	−0.003 (− 0.068 to 0.062)	0.927
Private sector (Reference)	0	
**Sufficiency of income for expenses**		
Completely	0.907 (0.784 to 1.026)	< 0.001
To some extent	0.759 (0.651 to 0.867)	< 0.001
Absolutely not (Reference)	0	

## Discussion

This was the first study into the HPB of Iranian men. The results showed that men moderately engaged in health-promoting behaviors. Social support had a direct significant correlation with the HPB; in addition, social support, age, educational attainment, marital status, job, and income sufficiency were the predictors of the HPB.

The mean total score of HPB was 2.72, which was consistent with a study on women at reproductive age [19], lower than a study on university students in Gilan, Iran [31], and higher than a similar study in Indonesia [32]. These results may be affected by Iranian culture, social environment, and participants’ characteristics.

Consistent with other domestic studies, men obtained the highest score in spiritual growth [33, 34]. This result is due to the cultural and religious status of society, as was mentioned in other studies [35, 36]. On the other hand, physical activity had the lowest score, which was confirmed by the majority of studies on the HPB in different groups [19, 34, 37, 38]. This is because men engage less than women in physical activities [39, 40]. Many factors are associated with a reduced tendency towards physical activities. Among these factors is a lifestyle change caused by changes in cultural and social factors, and the living environment in the modern world [41]. In this study, social support was significantly correlated with HPB and its subdomains. The literature results indicated the positive effect of social support on HPB [19, 42, 43]. Given that social support is a predictor of HPB, social support-based interventions should be considered in HPB programs. The social support perceived by individuals is influenced by socio-economic factors and the cultural status of society. Social injustice, especially in the racial and religious minorities, undermines perceived social support and self-efficacy, and increases the likelihood of developing physical and mental illness [44]. Across life periods, sources of social support are different, among children and adolescents, parental support is most important, whereas adults and older adults relied more on spouses, followed by family and then friends [45]. Among older adults, a formal source of social support with a more secure financial status has the most effect on HPB and life satisfaction [46]. Studies demonstrate that one’s social support network plays an important role in promoting healthy behaviors [43, 47, 48]. This issue especially in low-income areas such as Iran, that there are medical and human resource’s limitations for community healthcare systems, is very important.

Age is another predictor, which was significantly correlated with the total score of HPB. This finding was observed in other studies [43, 49–51]. People’s tendency towards the adoption of HPB increases with aging [41, 52, 53]. This finding indicates the positive effect of life experiences as external variables. In fact, lifestyle improvement in older people is associated with greater changes [54].

Job and income, as another predictors, were significantly correlated with the total score of the HPB. In this regard, the managers and experts and also the participants with sufficient income achieved the highest score. These findings were observed in other studies too [41, 55, 56]. Job not only directly affects men’s health [57–61] but also affect their access to healthy food, as well as welfare and healthcare facilities [62]. Insufficient income not only increases the level of stress but also the likelihood of unhealthy behaviors in men [56, 63]. In fact, job status and income are directly related to each other. Having a better job leads to more income. Studies have shown that having a better job position, and higher income reduce the risk of disease and people are more likely to engage in health-promoting behaviors. This finding may be due to better access to health-related services [64, 65].

According to the results, health promotion may be affected by many personal, cultural, and social factors. Therefore, it is needed to make appropriate interventions with further identification of facilitators and barriers to the adoption of the HPB. According to a qualitative study, men believed that they should have as much access to health resources as women do and barriers to such access should be eliminated. Contrary to general beliefs, men tend to cooperate with the healthcare staff and open up their needs with them [66]. Therefore, further studies, specifically qualitative studies, are needed to clarify men’s perception and experience of the HPB.

Among the strengths of this study was using a large sample size and random sampling technique. One research limitation is because of its cross-sectional nature, that the relationships observed between the HPB and demographic characteristics cannot be interpreted as a causality relationship.

### Recommendations

Based on the findings of this study, the following recommendations were suggested: 1- Since reduced physical activity has a direct correlation with the majority of chronic diseases, such as obesity, diabetes, and cardiovascular diseases [67, 68], further studies, including qualitative studies, should be conducted to identify why physical activity is less common among men and how they perceive barriers to HPB. The findings can be used to make specific programs to encourage men to become more physically active and provide them with the required facilities; 2- Policy-makers and health care providers, should actively collaborate with adults’ social network resources to promote healthy behaviors. Although interventions and programs to strengthen the social support network of at-risk individuals especially in poor communities should be developed [47, 48]; 3- With aging, the people’s needs in different activities are different from younger people. On the other hand, the majority of societies, including Iranian societies, are facing with global aging population phenomenon. Therefore, to facilitate and institutionalize HPB in older men, structural changes should be made in society to enable them to include such behaviors in their daily life program; 4- Government plans and interventions to fight poverty and create appropriate job opportunities for men can contribute to health promotion in men; and 5- Supervising the price management process by the government, creating appropriate job opportunities, and providing vulnerable groups with supportive packages can greatly help men solve their economic problems.

## Conclusion

According to the results, Iranian men moderately engaged in health-promoting behaviors. The participants obtained the highest and lowers scores in spiritual growth and physical activity, respectively. Although social support had a direct significant correlation with the HPB; in addition, social support, age, job, and income sufficiency were the predictors of the HPB. The results also confirmed the importance of social support and modifying demographic variables in the adoption of the HPB. Therefore policy-makers and health care providers, should actively collaborate to develop comprehensive interventions and programs in order to encourage men to improve health-promoting behaviors, especially physical activity, and to strengthen the social support network of at-risk individuals.

## Data Availability

Not applicable.

## Abbreviations

HPL: Health-promoting lifestyle
HPB: Health-promoting Behaviors
HPLP-II: Health-Promoting Lifestyle Profile II
PRQ: Personal Resource Questionnaire
GLM: General Linear Model
DALYs: Disability-Adjusted Life Years
ICC: Intra-Correlation Coefficient
